# Melanoma arising in a Giant congenital melanocytic nevus: two case reports

**DOI:** 10.1186/s13000-019-0797-1

**Published:** 2019-02-19

**Authors:** Tatiana S. Belysheva, Yana V. Vishnevskaya, Tatiana V. Nasedkina, Marina A. Emelyanova, Ivan S. Abramov, Kristina V. Orlova, Ludmila N. Lubchenko, Igor A. Utyashev, Marina B. Doroshenko, Lev V. Demidov, Mamed D. Aliev

**Affiliations:** 1 Federal State Budgetary Institution, “N.N. Blokhin Medical Research Center of Oncology” of the Ministry of Health of the Russian Federation, 115478 Kashirskoye shosse, 24, Moscow, Russian Federation; 2 Engelhardt Institute of Molecular Biology, Russian Academy of Sciences, Moscow, Russian Federation

**Keywords:** Giant congenital melanocytic nevus, Melanoma, Neurocutaneous melanosis, Genetic analysis, *NRAS* mutation, Infants

## Abstract

**Background:**

A giant congenital melanocytic nevus (GCMN) is found in 0.1% of live-born infants. If present, the lesion has a chance of about 6% to develop into malignant melanoma. Both children and adults can be affected by malignant melanoma arising in a giant congenital nevus. Up to 95% of GCMNs harbor *NRAS* mutations, and mutations in the *BRAF*, *MC1R*, *TP53*, and *GNAQ* genes have also been described. The individualization of therapy is required, but diagnostic and prognostic criteria remain controversial.

**Case presentations:**

We report two cases: 1) melanoma arising in a giant congenital nevus during the first month of life complicated with neurocutaneous melanosis (NCM), and 2) melanoma arising in a giant congenital nevus during the first 6 months of life. Pathology, immunohistochemistry, and genetic analyses of tumor tissue were performed. The first case revealed only a non-pathogenic P72R polymorphism of the *TP53* gene in the homozygote condition. For the second case, a Q61K mutation was detected in the *NRAS* gene.

**Conclusion:**

Malignant melanoma associated with GCMN is rare and therefore poorly understood. Outcomes have been linked to the stage at diagnosis, but no additional pathological prognostic factors have been identified. The most frequent genetic event in giant CMNs is NRAS mutations, which was discovered in one of our cases. To accumulate evidence to improve disease prognosis and outcomes, children with congenital melanocytic nevus should be included in a systemic follow-up study from birth.

## Background

Malignant melanoma is a rare tumor during childhood and accounts for up to 0.9% of all pediatric malignancies [1, 2]. A congenital melanocytic nevus (CMN) is clinically defined as a melanocytic lesion that is present at birth or develops during infancy from preexistent melanocytes [3–5]. CMNs typically affect the trunk and proximal parts of the limbs, scalp, and neck, but might involve any other skin surface. Congenital melanocytic nevi are usually classified by size. The risk of developing melanoma over a CMN is believed to be directly proportional to the size of the nevus and varies from 2.6 to 4.9% for small and medium nevi and from 6 to 20% for giant nevi [6]. There has been controversy about the incidence of melanoma and thus the clinical management of CMN, which is partly due to the difficulties of histological diagnosis and partly due to publishing bias towards cases of malignancy.

Giant congenital melanocytic nevus (GCMN) is usually defined as a melanocytic lesion that is present at birth and will reach a diameter of ≥20 cm in adulthood. Its incidence is estimated as < 1:20,000 newborns, of which about 6% develop melanoma at the site of the nevus. GCMN is the main risk factor for the development of melanoma in childhood. Currently, there is tremendous uncertainty regarding how GCMNs should be treated. The standard approach is based on two main considerations: (1) obtaining an acceptable cosmetic result to decrease the psychosocial inconvenience to the patient and (2) minimizing the risk of malignancy. However, there are descriptions of clinical cases where melanoma developed after the removal of a giant nevus [7] and in old age [8].

GCMN usually occurs sporadically [9, 10], but rare familial cases have also been reported [11, 12]. A report of monozygotic twins discordant for GCMN suggests that a postzygotic event might be involved [10]. The etiology and pathogenesis of GCMN are not fully understood. Various mechanisms have been posited, such as defects in neural crest development [13–15], activating mutations leading to uncontrolled melanocyte proliferation [16, 17], cutaneous mosaicism, and paradominant inheritance [12]. Melanoblasts originate from the neural crest cells, and their proliferation, migration, and differentiation are regulated by a complex network of interacting genes. Mutations in this network, such as in genes *MITF* and *KIT* and probably in the hepatocyte growth factor/c-Met signaling pathway, might deregulate the pigmentation system during embryogenesis, resulting in various congenital disorders [12].

Another gene network that controls the proliferation of melanocytes is the RAS/RAF/MAPK signaling pathway. Various activating mutations in this pathway have been identified, which involve the genes *NRAS* [16, 18], *BRAF* [18–20], and *GNAQ* [21]. Postzygotic mutations in the *NRAS* gene are thought to be responsible for CMN formation in 80% of cases because the same mutation is found in different cutaneous lesions from the same individual and in affected neurological and malignant tissue. The *NRAS* mutations often result in an amino acid substitution in codon 61. The *BRAF* V600E mutation can also be found but in no more than one lesion of the same patient and therefore cannot be assigned as causal [22]. Additionally, mutations in *MC1R* [18, 23] and *TP53* [18] have been identified in CMN and might be involved in its formation. The presence of *BRAF* or *NRAS* mutations does not confer an increased risk of malignant transformation [21], and further mutations are required to cause melanoma formation in a CMN [22].

Both children and adults can be affected by malignant melanoma arising in GCMN, which has a bimodal distribution with around 70% of cases occurring in childhood. Differential diagnosis should be done to distinguish between malignant and benign proliferations that may resemble malignant melanoma but usually lack progressive growth or ulceration. Benign proliferations within CMN are common and primarily arise in large or multiple nevi, although not exclusively. Knowledge of their features is helpful in monitoring for malignancy. In addition to proliferative nodules, GCMN are often associated with “satellite nevi,” which are smaller CMN that are present at birth or arise months to years later.

Only several large institutions have experience in treating children (from birth to 1 year old) with malignant melanoma. The outcomes described were linked to the stage at diagnosis and the presence of severe complications, such as neurocutaneous melanosis or melanoma in the central nervous system (CNS) [24]. Neurocutaneous melanosis (NCM) is a rare syndrome that is characterized by benign or malignant proliferated melanocytic nodules in the CNS and is associated with the presence of congenital melanocytic lesions.

To date, no absolute guidelines to treat the GCMN have been established, and therefore, the subject remains one of the most controversial issues in dermatologic surgery and dermatologic oncology. We describe two rare cases of CMN: a case of melanoma arising in GCMN during the first month of life complicated with NCM, and another case arising in GCMN during the first 6 months of life. The clinical, pathological, and genetic characteristics of these patients are described, which provide evidence about this rare disease and generate data needed for the establishment of individual diagnostic and prognostic criteria.

## Case presentations

### Case 1

**Fig. 1:**
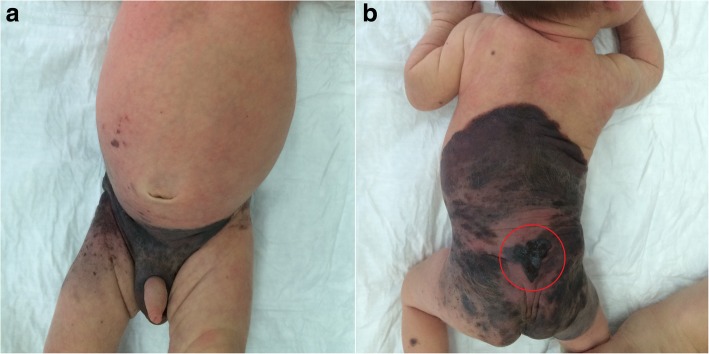
Patient M at 22 days old before surgery. **a** Front and (**b**) back views. A nodule was diagnosed in the lumbar-sacral part (marked with a red circle)

Patient M was referred to us at the age of 22 days. Written informed consent was obtained from the parents of the patient for publication of the case report and any accompanying images. This patient had skin phototype II according to the Fitzpatrick scale. The boy was born during the 37th week of pregnancy. The pregnancy had no complications, and there was no family history of melanoma or other types of cancer. The child’s parents reported a giant pigmented mole on the child’s skin that covered parts of the back and buttocks and extended to part of the pubis and the scrotum, which was revealed at birth. Within the pigmented mole, the parents also noted an anomalously formed center with a wet and bleeding surface in the lumbar-sacral part (Fig. 1).

**Fig. 2:**
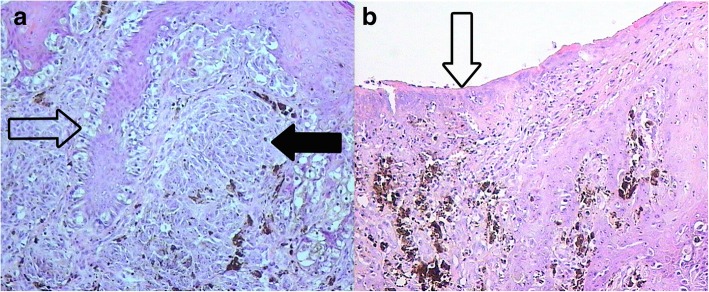
Cutaneous melanoma; hematoxylin and eosin staining. **a** Nodular melanoma. Polymorphous melanocytes in the basal layer of the epidermis (transparent arrow) in the dermis in the condition of nested clusters and fields (black arrow), × 10 magnification. **b** Ulceration on the surface of the tumor (arrow), × 10 magnification

**Fig. 3:**
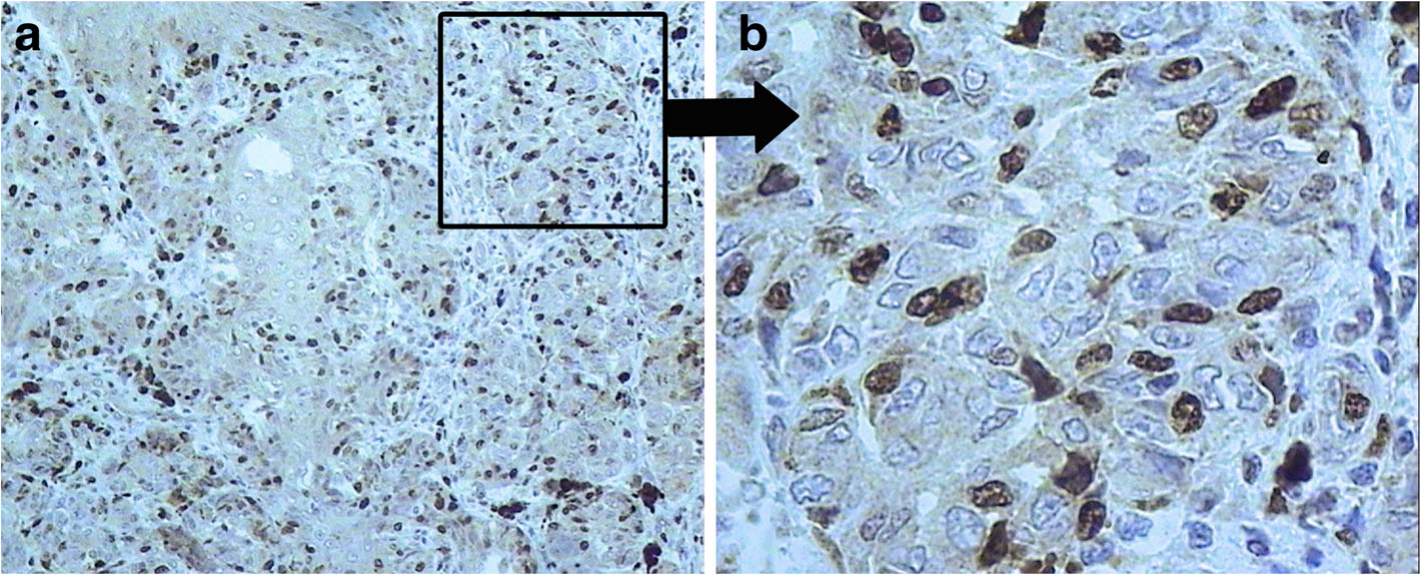
Cutaneous melanoma; immunohistochemical staining of Ki-67. **a** × 10 and (**b**) × 40 magnifications

Upon clinical examination, a giant pigment mole (larger than 20 cm) was observed in mostly the dorsal part of the body, which was partially covered with hair with irregular pigmentation. The colors varied from pink to dark-brown or black. A rapidly growing nodule with irregular forms (3.5 × 3.7 cm) of black color and ulceration was detected in the lumbar-sacral part (Fig. 1b). There were single satellites on the hair-covered part of the head, body, and extremities. A dermatoscopic exam was performed. Based on anamnesis, clinical investigation, and dermatoscopy, we diagnosed a giant congenital nevus with satellites, together with congenital malignant melanoma of the lumbar-sacral part with ulceration. Complete ultrasound examination of the lymph nodes, abdomen, retroperitoneal part, and pelvis was performed, as well as X-rays of the chest. Excision biopsy with local tissue plastic was performed under anesthesia in July 2015. Histopathology revealed a nodular epithelioid and nevoid-cell pigment-containing melanoma (Fig. 2a), which arose in the congenital nevus with 1 mitosis/mm^2^, ulceration (Fig. 2b), vertical growth phase, Clark invasion level 3, and Breslow thickness of 1.5 mm. There were no symptoms of vessel invasion. Immunohistochemistry was performed to detect the expression of melanocyte markers (Melan A, HMB 45), and the Ki67 proliferation index of the tumor cells was 20–30% (Fig. 3).

Genetic analysis of tumor tissue was performed using a diagnostic biochip (EIMB RAS, Russia) for the detection of most common somatic mutations in the *BRAF*, *NRAS*, *KIT*, *GNAQ*, *GNA11*, *MAP2K1*, and *MAP2K2* genes [25]. No mutations in the above-mentioned genes were detected. Additionally, next-generation sequencing was used for exon analysis of the *NRAS, PDGFRA, KIT, RASA1, RAC1, MET, BRAF, PTEN, AKT1, MAP2K1, MAP2K2, TP53*, and *TERT* genes. To identify germinal mutations, exons of the *CDKN2A* gene were analyzed by Sanger sequencing. As a result, only Arg/Arg polymorphism at codon 72 of the *TP53* gene, which is not pathogenic, was revealed.

**Fig. 4:**
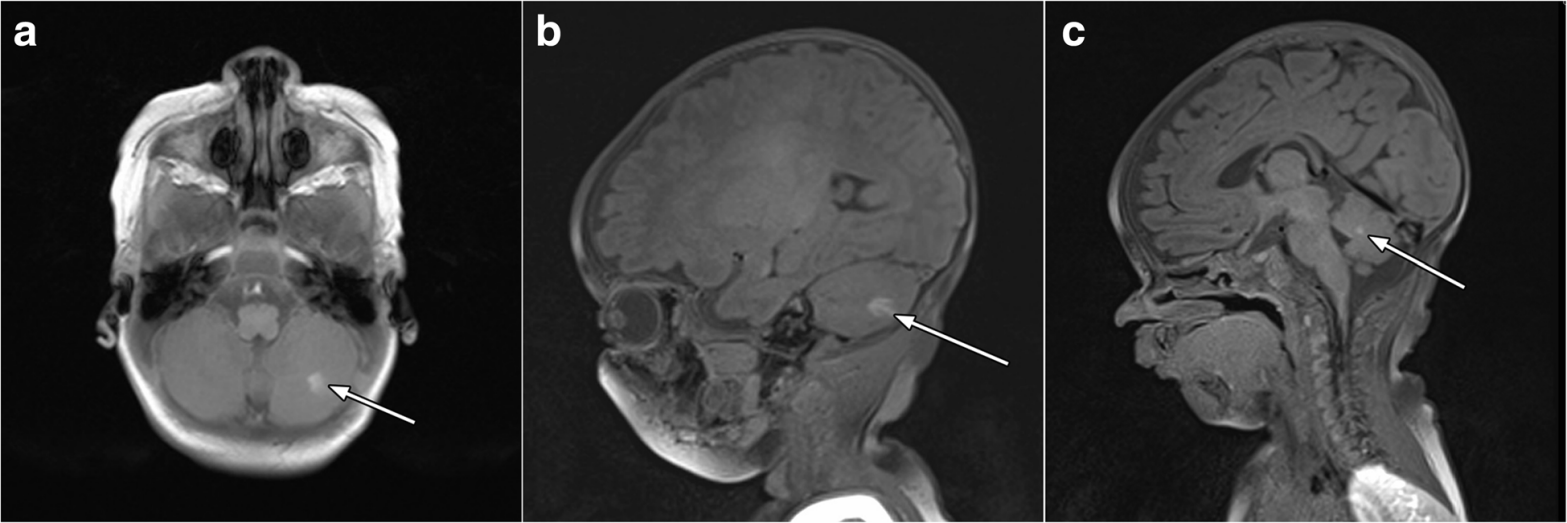
Magnetic resonance imaging shows the presence of melanin in the structure of the brain. **a** Postcontrast axial T1-weighted (W) MRI and (**b**) postcontrast sagittal T1W image with focus on the altered MR signal in the left hemisphere of the cerebellum is determined. **c** Sagittal T1W image with focus on the abnormal MR signal on the cerebral pia mater of the cerebellum. The imaging findings described were diagnostic for neurocutaneous melanosis

The presence of melanin in the brain structures was revealed by magnetic resonance imaging (MRI) of the whole body. The MRI of the melanin centers did not indicate melanoma metastasis and was typical for NCM (Fig. 4). This finding illustrates the importance of carrying out MRI for children with giant congenital nevus. The patient had no focal neurological symptoms such as seizures and no signs and symptoms of raised intracranial pressure. Based on pathology data and full examination of the patient, we made a diagnosis of melanoma T2bN0M0 stage IIA plus NCM, and dynamic observation was recommended. At present (June 2018), the child is under observation and shows no symptoms of disease progression. The focal leptomeningeal deposits are stable.

### Case 2

**Fig. 5:**
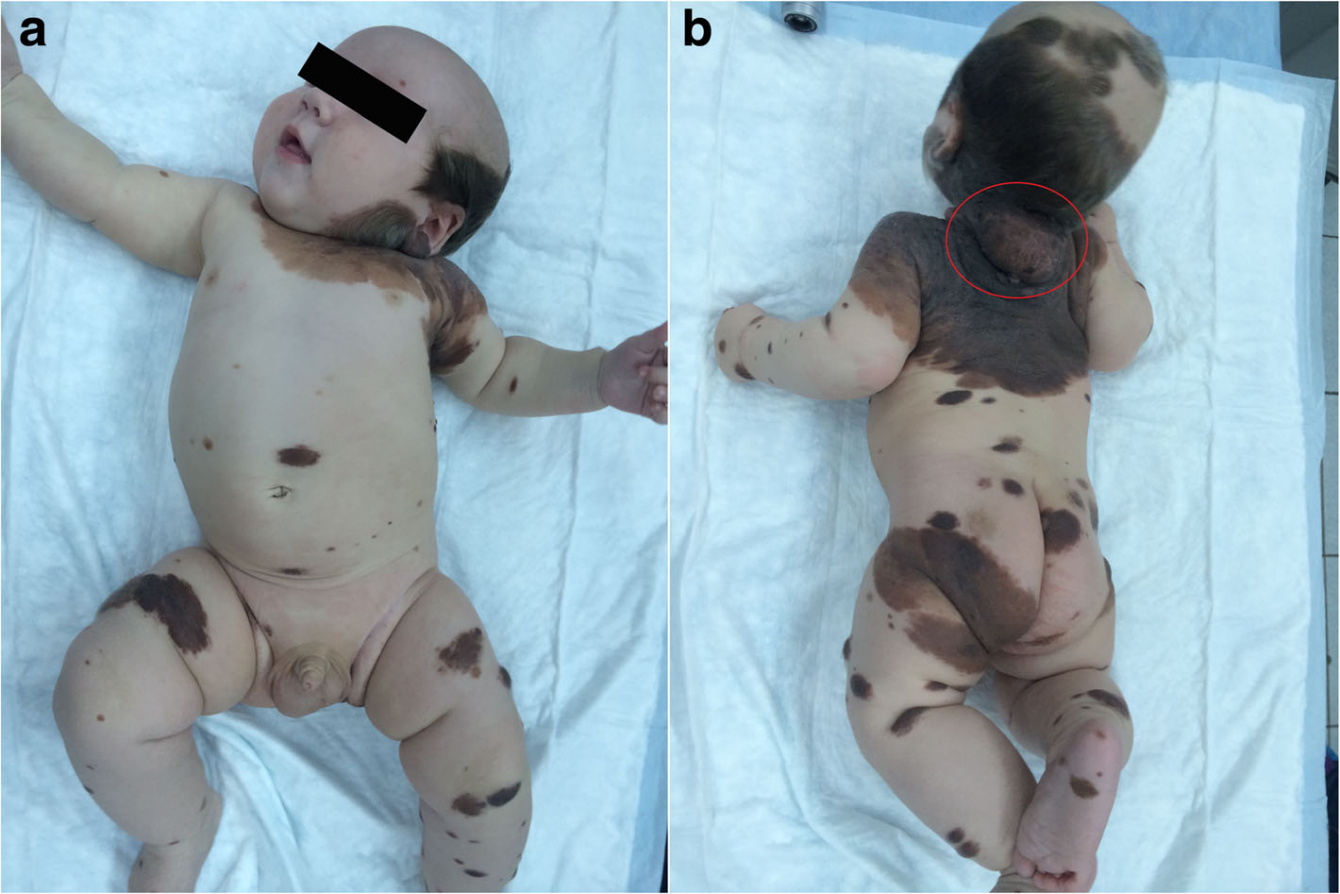
Patient L at 5 months old before treatment. **a** Front view and (**b**) back view

Patient L was 5 months old at referral. Written informed consent was obtained for publication of the case report with accompanying images. The patient’s skin was Fitzpatrick phototype II. The patient’s mother came with complaints about a giant pigmented mole that was present from birth. It was mostly located in an occipital part of the child’s head and extended to the back part of the neck, upper part of the shoulders, and the back and chest. There were also numerous isolated pigment masses on the child’s body and limbs (Fig. 5). The boy was born during the 39th week of pregnancy. There was a giant pigmented mole (larger than 20 cm) that was partially covered with hair and characterized by irregular pigmentation with colors from pink and light-brown to grey-black. The mole was seen mostly on the upper back and partially spread to the chest, back of the neck, and crown of the head. A large, newly pigmented neoplasm was detected on the back of the neck.

**Fig. 6:**
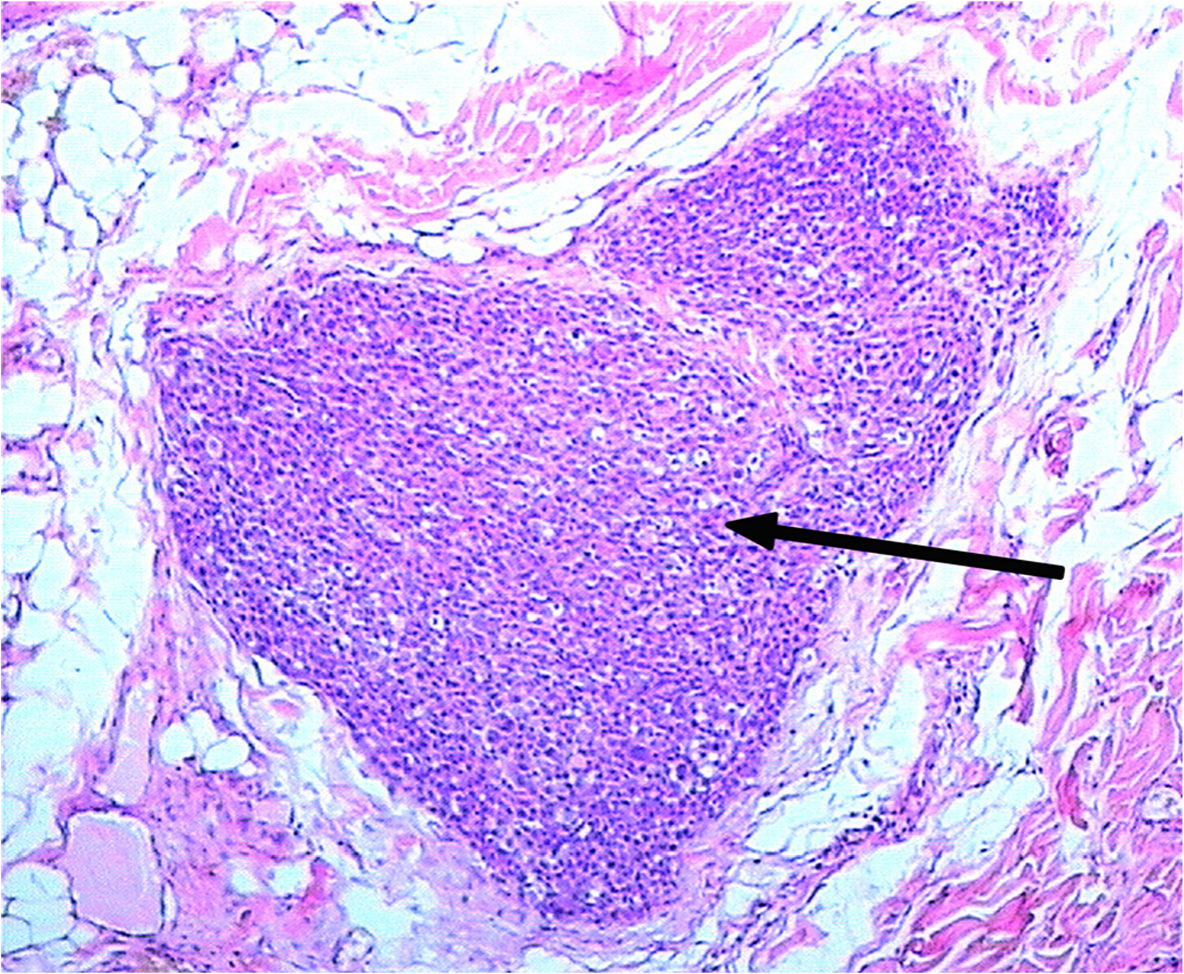
A melanoma satellite in the subcutaneous tissue. Hematoxylin and eosin staining, × 5 magnification

The dermatoscopic picture was not informative. Based on anamnesis and a clinical examination, we made a diagnosis of giant congenital nevus with satellites and congenital cutaneous melanoma of the back of the neck. Anesthetic was provided, and diagnostic excision biopsy was performed in October 2014. Ultrasound was used to examine the peripheral lymph nodes, abdomen, and retroperitoneal and pelvic organs. A СT scan of the chest and MRI of the neck were also performed. Ultrasound examination and MRI of the neck with intravenous contrast identified the metastatic satellite. Histopathology shows an epithelioid cell melanoma with 2 mitoses/mm^2^, satellites, and no ulceration or vessel invasion. The Breslow thickness without satellites was 2 mm. A distinctly shaped tumor node (indicated by an arrow in Fig. 6) was detected in the subcutaneous tissue and identified as a melanoma satellite (Fig. 6).

Genetic analysis of the tumor was performed using a diagnostic biochip for the detection of somatic mutations in the *BRAF*, *NRAS*, *KIT*, *GNAQ*, *GNA11*, *MAP2K1*, and *MAP2K2* genes, Sanger sequencing for searching germinal mutations in the *CDKN2A* gene. Eventually, a Q61K mutation was revealed in the *NRAS* gene.

Based on the pathology report and whole-body examination, we made a diagnosis of melanoma T2aN2cM0 stage IIIB. Observation was recommended because of the patient’s age. The child is currently (July 2018) under observation and shows no symptoms of disease progression.

## Discussion

Malignant melanoma is a rare neoplasm in pediatric patients, but children with CMN have a greater risk of melanoma. The size and location of the CMN and its association with multiple satellite nevi also seem to influence malignization and melanoma development. The risk of developing melanoma over a CMN is believed to be directly proportional to the size of the nevus [6]. According to the literature, 67% of cases have revealed primary melanoma within the nevus, with 14% showing metastatic melanoma with an unknown primary site and 8% showing extracutaneous melanoma [26]. Cutaneous melanoma arising in the CMN usually presents as a new nodule or lump that mainly arises in the deeper dermis or subcutaneous tissue [27, 28]. Most of the clinical reports (up to 90%) indicate malignant melanoma on the trunk [29]. Only isolated clinical cases or small series of cases are described, which confirm the rare occurrence and necessity of accumulating data and clinical experience [30]. Table 1 summarizes information about cutaneous melanoma cases arising in GCMN. Some of the reports contain only clinical and dermatoscopic characteristics with no detailed pathological and genetic data [8, 30, 31].

**Table 1:** Clinical and genetic features of patients with congenital melanocytic naevus (CMN) and melanoma: literature data and own experience

Author, Case №	Sex	Age at diagnosis (years)	Outcome/time after diagnosis	MRI CNS	Primary melanoma site	Tissue for genetic analysis	Genetics
Literature data							
Streams et al., Case 1 [7]	Female	44	Alive	Not done	Primary malignant melanoma of the left forearm underneath an intact skin graft 40 years after having had a partial excision and grafting of her GCMN	Not done	Not done
Tchernev et al., Case 1 [8]	Female	61	Alive	Not done	Malignant melanoma of the occipital region (stage IIB)	Not done	Not done
Lalor et al., Case 1 [35]	Female	8	Alive	Normal	Nodular melanoma on the scalp	Primary tumor	*NRAS* Q61R mutation
De la Rosa Carillo [9]		0 d	Died, 5mo	NCM	Large, multilobular, pigmented lesion covering 35% of the body, atypical melanocytic proliferation. Congenital melanoma.	Several biopsies	*NRAS* Wild-type, *BRAF* Wild-type
Kinsler et al., Case 4 [22]	Male	15, 5	Alive, 11 mo	Normal	Cutaneous, within largest CMN on the back of the scalp and neck, metastatic to local lymph node at time of diagnosis	Cutaneous melanoma	*NRAS* Wild-type
Kinsler et al., Case 10 [22]	Male	Not known	Death, age 2,4 years	Normal	Lymph node groin, locally recurrent despite excision, local metastasis	Not done	Not done
Kinsler et al., Case 12 [22]	Female	6,5	Death, 6 mo	Normal	Cutaneous, within largest CMN, at the site of postnatal resection of a benign congenital nodule, metastatic to local lymph node at time of diagnosis	Cutaneous melanoma	*NRAS* Q61K mutation
Maguire et al., Case 2 [31]	Female	7 mo	Alive, 9 mo	Not done	Cutaneous, on the groin site. A wide local excision was done. At 16 months of age enlarged node in the groin, metastatic melanoma	Not done	Not done
Own experience							
Case 1	Male	22 d	Alive	NCM	Congenital cutaneous malignant melanoma of the lumbar-sacral part	Primary tumor	*NRAS* Wild-type, *BRAF* Wild-type
Case 2	Male	5 mo	Alive	Normal	Congenital cutaneous malignant melanoma of the back of the neck	Primary tumor	*NRAS* Q61R mutation

We describe two cases of melanoma arising in a giant CMN as a new growing nodule and provide detailed clinical, dermatoscopic, pathological, and genetic analyses. When a diagnosis of cutaneous melanoma is suspected in a CMN patient, a biopsy should be performed (through excision if possible) with a detailed histopathological examination by at least two experts. Melanomas arising from giant congenital nevus predominantly develop from dermal melanocytes, as opposed to the melanomas emerging from small and medium nevi, which originate from the epidermis [3].

Clark reported that diagnostic problems at the histological level are due to the complex cellular composition of some of the nodular overgrowths occurring in congenital melanocytic lesions. Four major histological patterns of proliferation have been observed in CMN at birth or in the neonatal period: 1) simulants of superficial spreading melanoma with increased numbers of large epithelioid melanocytes; 2) simulants of nodular melanoma with black nodules of epithelioid melanocytes; 3) nodular proliferative neurocristic hamartomas; and 4) biologic malignant melanomas, which are mostly characterized by small “blastic” pleomorphic melanocytes with a high mitotic rate. Clark believed that the true melanomas with metastatic potential usually develop after the neonatal period [32]. Our cases confirm Clark’s findings because both children are now under observation without signs of disease progression despite the stage, early age of melanoma development, and subsequent surgical treatment.

Histological examination is the standard for the diagnosis of malignant melanoma and was performed in both of our cases. In the first case, the histopathology showed a nodular type of melanoma (Fig. 2a), which arose in the congenital nevus background with 1 mitosis/mm^2^, ulceration (Fig. 2b), vertical growth phase, Clark invasion level 3, and Breslow thickness of 1.5 mm, without any symptoms of vessel invasion. According to immunohistochemical analysis, the tumor cells were positive for melanoma-specific markers Melan A and HMB45.

Ki67 is frequently used as an indicator of cell proliferation, and the Ki67 index of proliferation was 20–30% in tumor cells (Fig. 3). According to the literature, the Ki67 index is not a good marker for prognosis or confirming malignancy in cases of GCN burdened by melanoma. The melanoma sometimes might be a mitotically active proliferative nodule arising in a GCMN. This feature is worrisome when encountered in melanocytic lesions, but by itself, it should not trigger a diagnosis of melanoma in the absence of other histologic criteria of malignancy [33]. We used Ki67 as an additional marker. In ***Case 2***, histopathology showed an epithelioid cell melanoma with 2 mitoses/mm^2^, ulceration, satellites, and no vessel invasion (Fig. 6). The Breslow thickness (without satellites) was 2 mm. The tumor node (melanoma satellite) was determined in subcutaneous tissue (Fig. 6).

Molecular analysis was performed on the biopsies obtained, and several genes that might play a role in GCMN development and malignant transformation were investigated. For patient M (***Case 1***), we evaluated the presence of somatic and germline mutations in the *BRAF*, *NRAS*, *KIT*, *GNAQ*, *GNA11*, *MAP2K1/2, PDGFRA, RASA1, RAC1, MET, PTEN, AKT1, TP53*, and *TERT* genes and somatic mutations in the *GNAQ* and *GNA11* genes. Germline mutations in the *CDKN2A* gene were also studied. Only non-pathogenic *TP53* codon 72 Arg/Arg polymorphism was detected. For patient L ***(Case2)***, an analysis was performed to determine the somatic mutations in the *BRAF*, *NRAS*, *KIT*, *GNAQ*, *GNA11*, *MAP2K1*, and *MAP2K2* genes and germline mutations in the *CDKN2A* gene. Ultimately, only a Q61K mutation in the *NRAS* gene was found.

Mutations in the *NRAS* gene occur in 80–95% of giant CMNs [10, 34] and are considered one of the causes of CMN formation [22], although other factors are needed for malignant transformation [21]. In both of our cases, no specific mutation was identified that could account for the melanoma. Our colleagues previously published a clinical case of an 8-year-old girl with melanoma arising within a medium-size congenital nevus with NRAS Q61K mutation (case 5, Table 1) [35]. Kinsler et al. published the results of 25 years of experience at their center with melanoma in congenital melanocytic nevi, including the molecular characteristics of the tumor [23]. Among 12 patients with melanoma, 6 had melanoma in CNS, 3 had an unknown melanoma site, and 3 had cutaneous melanoma (Tables 1, 4–6). There were 9 patients who developed melanoma in the first 5 years of life, and 7 patients revealed a Q61K mutation in the NRAS gene. In 3 cases of cutaneous melanoma in one patient, Q61K was found as in our Case 2. When the diagnosis of cutaneous melanoma arising in a CMN is clinically suspected, an urgent biopsy should be performed (excision if possible) with histopathological examination by at least two experts. *NRAS* and *BRAF* hotspot genotyping by sensitive methods are recommended to improve diagnostic accuracy and guide management.

The treatment of CMN is one of the most complicated areas of surgical and dermatologic oncology, and there are no commonly accepted standards to treat this lesion. It is not clear whether it is necessary to remove a CMN to reduce the risk of melanoma. In some reports, despite the almost complete removal of the GCN, the surgery failed to prevent the development of malignant melanoma, and the role of surgical excision of GCN remains controversial [7, 36]. Although surgery does not reduce the risk of extracutaneous melanoma, the removal of melanocytic cells appears to reduce the risk of developing melanoma within the nevus. Some surgical options described for GCMN treatment include serial resection, skin grafts, and the use of tissue expanders [37].

Melanomas in the GCN usually develop before puberty in the first 5 years of life, as opposed to the small and medium nevi, where it routinely occurs after puberty [4, 5, 38].

NCM is a rare complication that worsens the prognosis of GCN patients. NCM is neuromelanosis associated with CMN, which describes melanocytic proliferation (benign or malignant and nodular or diffuse) within the leptomeninges and brain parenchyma [4–6, 9–11]. Kadonaga described NCM and redefined it as the presence of a CMN larger than 20 cm or multiple CMN (more than three) in association with meningeal melanosis or melanoma [39]. Our first case had NCM and malignant melanoma arising in a GCN larger than 20 cm. Malignant melanoma and NCM most often occur in patients with CMN that have a diameter > 40 cm, multiple satellite nevi, and a truncal location. Almost one-third of all patients with NCM have numerous medium-sized CMN.

For individuals at risk of NCM who are younger than 6 months old, gadolinium-enhanced screening MRI is recommended for long-term neurological observation [4]. Patients with neuromelanosis may be symptomatic or asymptomatic. In our case, MRI revealed the presence of melanin in the structures of the brain, which was performed despite an absence of neurological symptoms. Therefore, MRI evaluation should be performed for newborns with GCN (and especially with multiple satellite nevi).

The suggested work-up for a patient with CMN and a confirmed diagnosis of melanoma is the following: (1) full blood count and lactate dehydrogenase level; (2): CNS MRI with gadolinium contrast, whole-body positron emission tomography–computed tomography scan or computed tomography scans; (3) tissue sample for histopathology, *NRAS,* and *BRAF* hotspot genotyping and copy-number analysis (array CGH or SNP array or FISH). The genotyping may be important to therapy strategies including use of mitogen-activated protein kinase kinase (MEK) inhibitors in NRAS-mutated tumours [40].

## Conclusion

Malignant melanoma arising in a GCNM is rare and poorly understood. Children with CMN should be included in a systemic follow-up from birth. The histopathology by at least two experts in the field and genetic analysis of driver mutations can help to differentiate melanoma from benign proliferative nodules in the skin. The most frequent genetic event in giant CMNs are *NRAS* mutations (up to 95%), which was discovered in one of our cases. Given the rarity of the disease, it is important to accumulate new evidence concerning diagnostic features, prognosis, and clinical outcome.

## Abbreviations

CCM: Congenital cutaneous melanoma
CM: Cutaneous melanoma
CMN: Congenital melanocytic nevus
GCN: Giant congenital nevus
MRI: Magnetic resonance imaging
NCM: neurocutaneous melanosis
